# Thoracoabdominal Asynchrony in Healthy Children

**DOI:** 10.1111/jsr.70394

**Published:** 2026-06-18

**Authors:** Kristjan Dereksson, Sigrun Helga Lund, Agneta Markström, Sigridur Sigurdardottir, Erna Sif Arnardottir, Michael V. Clausen, Sigurveig T. Sigurdardottir, Tryggvi Helgason, Erik A. Eklund

**Affiliations:** ^1^ Pediatrics, Clinical Sciences Lund University Lund Sweden; ^2^ Children‘s Hospital Reykjavik Landspitali University Hospital Reykjavik Iceland; ^3^ School of Engineering and Natural Sciences University of Iceland Reykjavik Iceland; ^4^ Department of Women's and Children's Health Karolinska Institutet Stockholm Sweden; ^5^ Reykjavik University Sleep Institute, School of Technology Reykjavik University Reykjavik Iceland; ^6^ Department of Allergy and Clinical Immunology Landspitali University Hospital Reykjavik Iceland; ^7^ Faculty of Medicine University of Iceland Reykjavik Reykjavik Iceland

**Keywords:** obesity, obstructive sleep apnea, polysomnography, respiratory effort, snoring

## Abstract

Measuring asynchrony between the thorax and abdomen during sleep provides information about inspiratory effort and upper airway obstruction in children, aiding in the diagnosis of obstructive sleep apnea (OSA). This study aimed to define a normative range for thoracoabdominal asynchrony (TAA) in healthy children by studying polysomnographies of 113 children aged 10–13 years. The percentage of total sleep time (TST) spent with elevated TAA, > 40°, was measured, as well as obstructive apnea‐hypopnea index (OAHI), sleep position, sleep stage and snoring. Subjects without measurable OSA (*N* = 94) were defined as healthy subjects and they had elevated TAA for an average of 3.3% of their TST while subjects with OSA (*N* = 19) had elevated TAA for 10.7% of their TST (*p* < 0.00001). The cut‐off point for the normal prevalence of elevated TAA was estimated at 9.6% of TST, which can be rounded up to 10% for ease of use. TAA prevalence rose in REM‐sleep and in the supine position and was related to increased snoring. Children with obesity had higher OAHI, more prevalent TAA and more snoring. No gender differences were observed. Parent‐reported PSQ scores showed limited concordance with objective measures. TAA assessment, available from standard RIP‐bands placed around chest and abdomen, may aid in the interpretation of sleep studies in children, both home sleep apnea tests and polysomnographies. With further research, they might help guide clinical decision making in paediatric sleep medicine.

**Abbreviations**
AASM: American Academy of Sleep Medicine
AHI: apnea‐hypopnea index
BMI: body mass index
CI: confidence interval
EEG: electroencephalography
EMG: electromyography
HSAT: home sleep apnea test
OAHI: obstructive apnea‐hypopnea index
OSA: obstructive sleep apnea
PSG: polysomnography
PSQ: paediatric sleep questionnaire
RDI: respiratory disturbance index
REM: rapid eye movement
RERA: respiratory‐effort related arousal
RIP: respiratory inductance plethysmography
SD: standard deviation
SDB: sleep disordered breathing
TAA: thoracoabdominal asynchrony
TST: total sleep time

## 1 Introduction

Obstructive sleep disordered breathing (SDB) in children ranges from mild primary snoring to upper airway resistance syndrome and obstructive sleep apnea (OSA). Severity correlates with increasing upper airway resistance, leading to heightened respiratory effort, flow limitation, and disrupted sleep (Trudo et al. 1998; Sankari et al. 2017).

Diagnosing SDB and OSA in children has historically been challenging due to difficulties defining pathological sleep‐related breathing and finding the best practical measurement methods. The gold standard for diagnosing OSA, in‐lab polysomnography (PSG), is also the only accepted method for ruling out OSA in children (Kirk et al. 2017). However, PSG is resource‐intensive and can be distressing for children and parents, and the method itself can disturb normal sleep. Many paediatric centres lack immediate access to PSG, fuelling interest in simpler, cost‐effective, home‐based diagnostic options, with or without EEG sleep staging (Sigurdardottir et al. 2025; Alonso‐Alvarez et al. 2015).

The correlation between PSG‐defined obstructive events and sleep related morbidity in children has been questioned (Gozal and Kheirandish‐Gozal 2010) as has the reliance on the apnea‐hypopnea index (AHI) as the primary disease metric (Punjabi 2016). One alternative is the respiratory disturbance index (RDI) which accounts for both AHI and respiratory effort related arousals (RERAs), and has been found to more accurately define symptomatic SDB than AHI alone (Guilleminault et al. 2004; Berry et al. 2012). Similarly, respiratory arousal events in PSG studies correlate with increased respiratory effort (Vincken et al. 1987; Gleeson et al. 1990) and it is reasonable to assume that correctly defining and measuring respiratory effort is an important part of defining the true impact of SDB in children. A comprehensive approach, combining parental reports (snoring, apneas, sleep disturbances, behavioural issues), apnea/hypopnea events, and direct respiratory effort measurements, may provide a more accurate assessment of SDB severity in children (Gozal and Kheirandish‐Gozal 2010; Guilleminault et al. 2004).

Respiratory effort can be assessed non‐invasively using respiratory inductance plethysmography (RIP), as is recommended by the American Academy of Sleep Medicine (AASM) (Berry et al. 2012) although lack of standardization hampers wider clinical use of such assessment. RIP‐bands are placed over the thorax and the abdomen and measure the expansion and contraction of those compartments associated with respiration. Using this method, a temporal relation between the movement of the two compartments can be established and a phase angle between them calculated (Carry et al. 1997; Hammer and Newth 2009) (Figures 1 and 2). During unlaboured breathing, the thoracoabdominal movements are synchronous, moving in the same direction at the same time, and having a degree of difference between their phase angle approaching 0°. Thoracoabdominal asynchrony (TAA) refers to a temporal lag between thoracic and abdominal motion, shown by a divergence in their movement at each point in time, with higher degrees signifying increasingly more pronounced asynchrony. The term “paradoxical breathing” is when TAA is at its most extreme and the abdomen is moving fully opposite to the thorax, showing a 180° TAA vector (Figure 1). A threshold of 40° TAA is used clinically to define elevated TAA in several countries, including Iceland, and was employed in this study.

**FIGURE 1:**
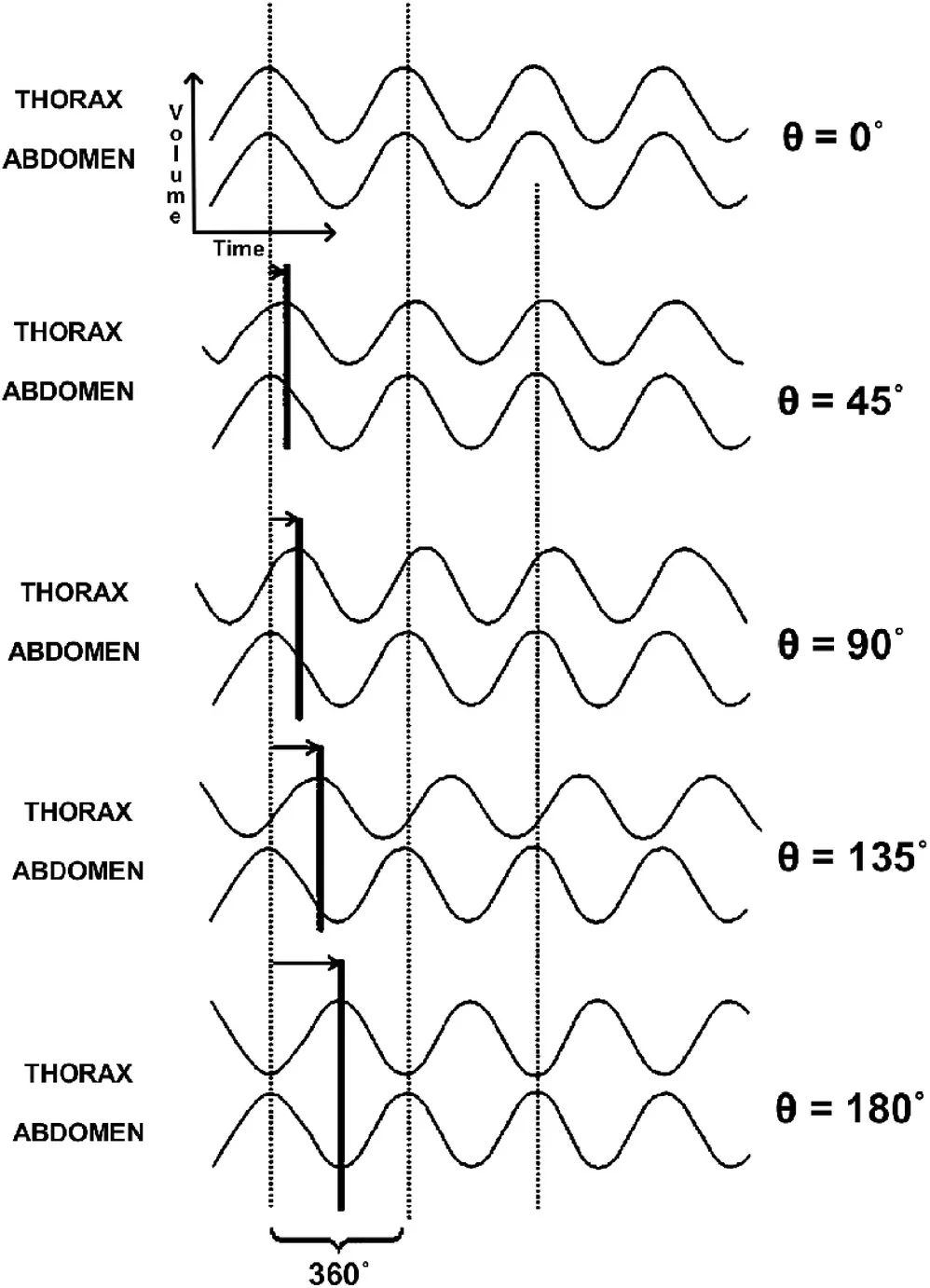
**Thoracoabdominal motion, shown with sine waves and the phase angle (*θ*) between the thorax and the abdomen.** Sine waves show the motion of the thorax and abdomen as changes in volume (*y* axis) over time (*x* axis). The maximal thoracal movement is shown relative to the maximal abdominal movement, with increasing asynchrony from top to bottom in the picture. Total synchrony of the motion of the thorax and abdomen has a phase angle (*θ*) of 0° while complete asynchrony (or paradoxical breathing) is reached at 180°, with opposite movement of the thorax and abdomen. The calculation of thoracoabdominal asynchrony has traditionally been done by phase angle analysis of a Lissajous curve, utilizing the method of Agostoni and Mognoni (Hammer and Newth 2009; Agostoni and Mognoni 1966). Adapted from Hammer and Newth (2009), with permission of Hammer, J. 2009.

**FIGURE 2:**
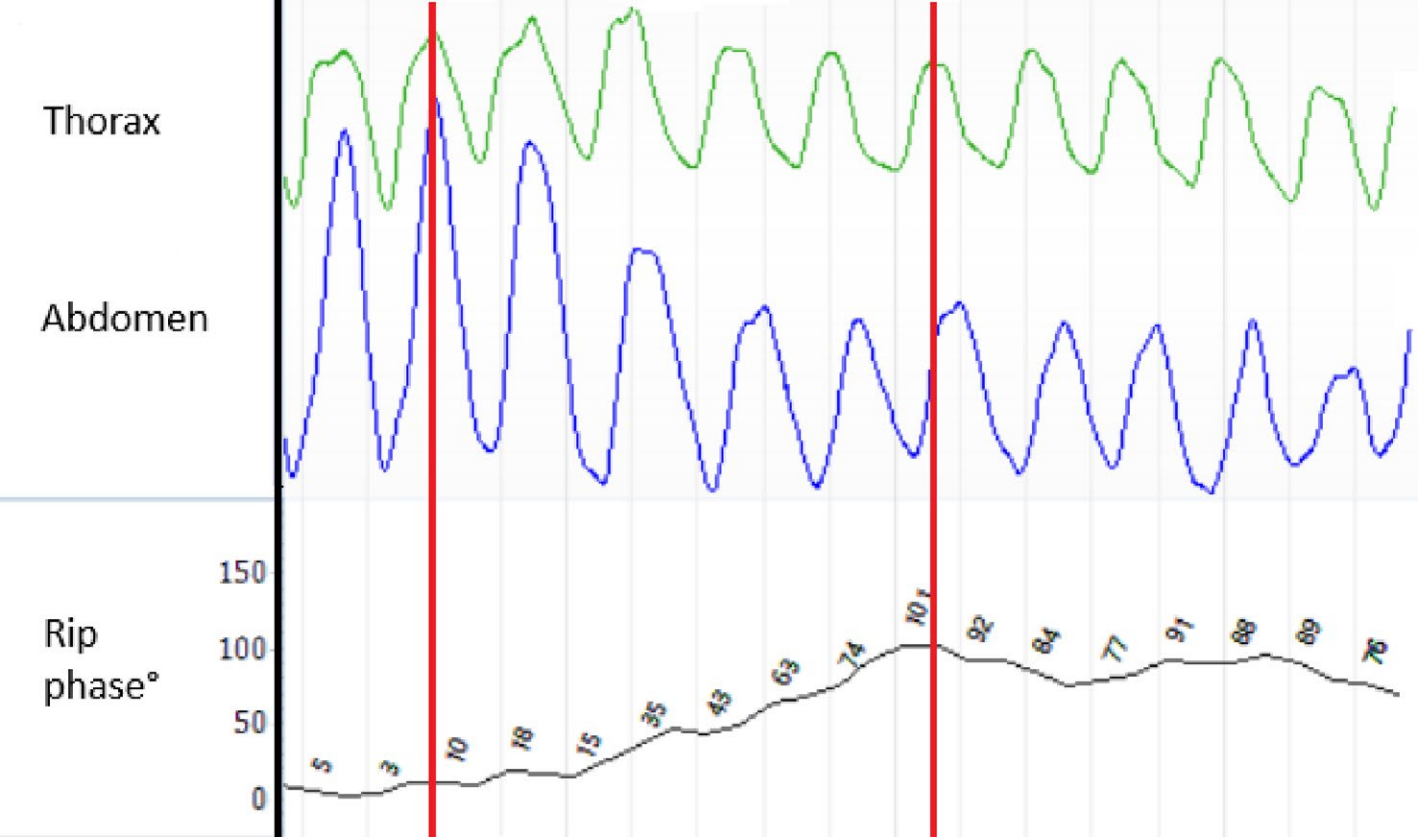
**Synchronous and asynchronous breathing.** The movement of the thorax and abdomen in relation to one another and the momentary RIP Phase angle (or thoracoabdominal asynchrony) calculated from those movements. The left vertical line marks synchronous breathing and the right vertical line marks asynchronous breathing, calculated as 101°.

TAA is generally a sign of increased inspiratory effort and relates to decreasing tidal volume (Hammer and Newth 2009). It is influenced by several factors such as upper airway resistance and collapsibility, as well as by lung‐ and chest‐wall compliance and respiratory muscle strength (Hammer and Newth 2009; Liu et al. 2017; Diaz et al. 1993). Increased TAA correlates with increased upper airway obstruction and OSA in children and adults (Immanuel et al. 2015; Marcus et al. 1994). It has been shown to be elevated during sleep periods even when apnea/hypopnea events are not detected (Immanuel et al. 2015), suggesting that AHI alone might underestimate actual obstruction. Post‐treatment improvement of TAA following adenotonsillectomy supports its potential as an objective marker of airway patency (Liu et al. 2017; Immanuel et al. 2015). TAA generally increases during rapid eye movement (REM) stage of sleep as well as in the supine sleep position (Liu et al. 2017; Immanuel et al. 2015; Hudgel et al. 1984). REM‐stage related increase of TAA is especially marked in infants, with a more compliant chest wall. This has been shown to decrease over the first 3 years of life, after which children and adolescents have similar levels of TAA as adults (Gaultier et al. 1987; Kohyama et al. 2001; Kohyama et al. 2000).

A certain amount of TAA is observed in both children and adults without SDB but the threshold distinguishing normal from pathological TAA in children remains undefined. Although several methods have been suggested, there is currently no consensus on the methods for measuring or quantifying normal TAA in children (Kohyama et al. 2000; Choi et al. 2020; Bronstein et al. 2018).

The primary goal of this study was to establish a normative TAA range for healthy 10‐ to 13‐year‐old children without OSA. This range could then be compared to measured TAA in children of the same age with OSA. We also made a comparison of TAA with regards to gender, obesity, sleep position, and sleep stage, as well as snoring in our subjects.

Furthermore, our aim is that the definition of normal TAA could subsequently be used with existing methods to create a more complete clinical diagnostic tool for quantifying upper airway obstruction and SDB severity in children.

## 2 Material and Methods

### 2.1 Participants

The study subjects were 118 Icelandic children, aged 10–13 years. They were recruited from the EuroPrevall/iFAAM birth cohort study in Iceland that recruited newborns in 2005–2010 (Grabenhenrich et al. 2020; Sigurdardottir et al. 2021). As part of that long term cohort study, parents of 948 children completed an online questionnaire regarding snoring and apneic episodes in 2017, 2 years prior to this study. All children with reported snoring at least three nights per week and/or witnessed apneas at least once per week (*n* = 109) were invited to take part in this study, of which 55% (*n* = 60) accepted. In addition, 99 age matched subjects from the same cohort, without snoring or witnessed apneas were invited, of which 59% (*n* = 58) accepted. Four subjects were excluded due to insufficient study data for analysis, and one subject withdrew consent, leaving 113 study subjects (Table 1).

**TABLE 1:** Demographics of the study population.

*N* (male %)	113 (67%)
Age in years (±SD)	11.8 (± 0.8)
BMI (±SD)	20.5 (± 3.6)
*N* underweight	0
*N* normal‐weight (%)	71 (63%)
*N* overweight (%)	23 (20%)
*N* obese (%)	19 (17%)

Abbreviations: BMI, body mass index in kg/m^2^; SD, standard deviation.

Medical conditions directly associated with SDB (e.g., upper airway hypotonia, midfacial anomalies or micrognathia) were considered exclusion criteria. Each subject's medical history was reviewed by a paediatric specialist, and no cases meeting these criteria were identified.

The study was approved by the Ethical committee of Landspitali University Hospital in Iceland as well as the National Bioethics Committee of Iceland (VSN 18‐206). An informed written consent was obtained from a legal guardian of all subjects as well as oral consent from the subjects themselves.

### 2.2 Data Collection

The study was conducted at the Icelandic Children's Hospital (Reykjavik, Iceland) in 2019. Legal guardians answered a questionnaire about sleep habits and snoring, including the Paediatric Sleep Questionnaire (PSQ) to screen for possible SDB (Chervin et al. 2000). Height and weight were measured with standardised equipment at the Children's Hospital. The subjects were fitted with sleep study equipment (Nox A1, from Nox Medical, Reykjavik, Iceland) by experienced sleep technicians, both with a standardised PSG setup, an extra multichannel frontal EEG and a combined oronasal cannula. The extended equipment was due to a secondary study protocol comparing different PSG modalities, published recently (Sigurdardottir et al. 2025). Our study used only data from the standard PSG setup, according to the AASM protocol (Berry et al. 2012), regarding EEG, EOG, ECG, leg EMG, thoracic and abdominal RIP bands, oximeter and accelerometer. An oronasal pressure transducer was used for flow measurement. Subjects slept one night at home with the equipment.

### 2.3 Data Analysis

The PSG studies were scored according to the AASM paediatric scoring rules, version 2.5 (Berry et al. 2018), by two European Sleep Research Society‐certified sleep technicians, blinded to clinical data. Analyses were performed using Noxturnal^R^ (version 5.1.3, Nox Medical, Reykjavik, Iceland).

PSG data used for analysis in this study was as follows: AHI and obstructive apnea‐hypopnea index (OAHI, which excludes central apneas from the AHI score), sleep position (supine and non‐supine), sleep stage (REM and non‐REM), snoring and TAA. A minimum of 6 h of sleep data per subject was required as per AASM recommendation.

TAA degree was calculated by the Noxturnal^R^ program as the phase angle between thoracal and abdominal movement, at each moment. Three levels of increasing TAA were defined: > 40°, > 80° and > 120°, reported as a percentage of total sleep time (TST). > 40° asynchrony was chosen as the definition of elevated TAA due to being the default clinical standard in Noxturnal^R^. The values of 80° and 120° were chosen as clean multiples of that value, with a higher degree meaning more pronounced asynchrony between the motion of thorax and abdomen (Figure 1).

The normal, or healthy, amount of TAA in our study was obtained from the subjects with a normal amount of obstructive events, that is, with an OAHI < 1. Sorting by prevalence of TAA during TST, the lower 95% in this group was used to define the 95th percentile cut‐off for normal TAA during sleep. All subjects below that cut‐off were defined as ‘Normal TAA’ and those above as ‘Abnormal TAA’.

BMI percentiles by age and gender were used to categorize subjects into three subgroups: normal‐weight, overweight and obese. Subgroups were defined according to International Obesity Task Force (IOTF) guidelines and BMI charts (Cole et al. 2000).

PSQ questionnaires were scored with each positive answer giving 1 point, up to a maximum of 22 points. Total score is given as a ratio of positive answers to the total.

### 2.4 Statistical Analysis

Comparison between subgroups was done using an unpaired student's *t*‐test, while comparison within subjects was done using a paired student's *t*‐test. Statistical significance was set at *p* < 0.05.

## 3 Results

### 3.1 Defining Normal Thoracoabdominal Asynchrony

Of the 113 subjects, 90 (80%) had an AHI < 1 and 94 (83%) had an OAHI < 1. The latter group, having no measurable upper airway obstruction by AASM standards, was defined as healthy with regards to OSA.

In the OAHI < 1 group, the mean value for TAA of > 40° was 3.3% of TST. The mean value for TAA > 80° was 0.8% of TST, and for > 120° it was 0.3% of TST.

The 95th percentile cut‐off for > 40° TAA was 9.6% of TST, defining the normal prevalence of > 40° TAA. Similarly, the cut‐off was 2.7% and 0.9% for > 80° and > 120° TAA, respectively (Table 2).

**TABLE 2:** Thoracoabdominal asynchrony as a percentage of total sleep time.

Level of TAA	OAHI < 1, Mean (95% CI)	OAHI ≥ 1, Mean (95% CI)	Significance	OAHI < 1, 95th percentile cut‐off
> 40° in TST	3.3% (2.6–4.0)	10.7% (5.0–16.3)	*p* = 0.000002	9.6%
> 80° in TST	0.8% (0.6–1.0)	2.7% (1.2–4.2)	*p* = 0.000002	2.7%
> 120° in TST	0.3% (0.2–0.4)	0.9% (0.3–1.4)	*p* = 0.0001	0.9%

*Note:* The table shows comparison between the TAA of subjects with OAHI > 1 versus subjects with OAHI of 1 or higher. Values are % of total sleep time. Significance calculated with unpaired *T*‐test. The rightmost column shows the 95th percentile of TAA, as a % of total sleep time, of the OAHI < 1 group defined as healthy with regards to obstructive sleep apnea.

Abbreviations: CI, confidence interval; OAHI, obstructive apnea‐hypopnea index; TAA, thoracoabdominal asynchrony; TST, total sleep time.

### 3.2 Effects of Elevated Obstructive Apnea‐Hypopnea Index

Nineteen of the 113 subjects (17%) in this study had OSA, with elevated OAHI, ranging from 1.0 to 6.3. This group, OAHI ≥ 1, had significantly more TAA as % of their TST across all three TAA levels (> 40°, > 80° and > 120°) compared with the group without OSA (Table 2).

The OAHI ≥ 1 group's mean value for > 40° TAA was 10.7%, above the OAHI < 1 group's 95th percentile cut‐off value of 9.6% (Table 2).

Looking at > 40° TAA and using the 95th percentile cut‐off of 9.6% of TST, the 113 subjects could be divided into a “normal TAA” group (*N* = 102) and an “abnormal TAA” group (*N* = 11) with values below or above the cut‐off, respectively.

Comparison between TAA groups showed significantly higher OAHI for the abnormal TAA group (Table 3). The same held true for TAA > 80° and TAA > 120° (Table S1).

**TABLE 3:** Symptoms of sleep disordered breathing. Comparison of subjects with normal and abnormal thoracoabdominal asynchrony, using the 95th percentile cut‐off for 40° thoracoabdominal asynchrony.

	Normal TAA (*N* = 102)	Abnormal TAA (*N* = 11)	Significance
OAHI (95% CI)	0.5 (0.4–0.6)	1.5 (0.4–2.7)	*p* = 0.00001
Snore in TST (95% CI)	1.9% (1.1–2.8)	8.1% (−1.3–17.4)	*p* = 0.002
Snore in supine sleep (95% CI)	3.1% (1.6–4.6)	11.0% (−1.5–23.5)	*p* = 0.009

*Note:* The table shows comparison between the group of subjects with normal and abnormal amounts of > 40° TAA, as defined by the 95th percentile cut‐off. Significance between groups calculated with unpaired *T*‐test.

Abbreviations: CI, Confidence interval; OAHI, obstructive apnea‐hypopnea index; TAA, thoracoabdominal asynchrony; TST, total sleep time.

### 3.3 Relation Between Snoring and Thoracoabdominal Asynchrony

The mean amount of snoring as a percentage of TST was 2.5% ± 6.4% for the whole group. Snoring was more prevalent in the supine position than in the non‐supine position, at 3.8% ± 9.6% (*p* = 0.002 within‐subject comparison). Snoring in the OAHI < 1 group was present for 1.5% ± 3.5% of TST but 7.6% ± 12.3% in the OAHI ≥ 1 group (*p* = 0.0001).

Dividing the subjects into normal and abnormal TAA groups using the 95th percentile cut‐off for > 40° TAA showed significantly more prevalent snoring in the abnormal TAA group. The same was seen for snoring in supine sleep specifically (Table 3).

### 3.4 Effects of Supine Sleep Position and REM Sleep

At an individual level there was significantly more TAA in supine sleep as well as in REM sleep compared with in TST, at all three TAA levels. Comparing the OAHI < 1 and OAHI ≥ 1 groups, there was a significant difference across all three TAA levels in both supine sleep and REM sleep (Table 4).

**TABLE 4:** Thoracoabdominal asynchrony in supine‐ and REM sleep, presented as percentage of sleep time.

	OAHI < 1, Mean (95% CI)	OAHI ≥ 1, Mean (95% CI)	Significance
TAA in supine sleep			
> 40°	5.1% (3.9–6.3)*	15.0% (7.1–23.0)*	*p* = 0.00002
> 80°	1.3% (1.0–1.6)*	4.1% (1.2–7.0)*	*p* = 0.0001
> 120°	0.4% (0.3–0.6)*	1.1% (0.5–1.8)*	*p* = 0.0004
TAA in REM sleep			
> 40°	10.9% (8.5–13.2)*	19.5% (9.6–29.4)*	*p* = 0.01
> 80°	2.4% (1.6–3.1)*	6.6% (2.0–11.3)*	*p* = 0.001
> 120°	0.7% (0.4–0.9)*	2.9% (0.2–5.5)*	*p* = 0.001

*Note:* The table shows comparison between the TAA of subjects with OAHI less than 1 and subjects with OAHI of 1 or higher. Significance between groups calculated with unpaired *T*‐test.

Abbreviations: CI, confidence interval; OAHI, obstructive apnea‐hypopnea index; REM, rapid eye movement; TAA, thoracoabdominal asynchrony; TST, Total sleep time.

* Significant at *p* < 0.05 by paired *T*‐test, compared to TAA in TST.

### 3.5 Effects of Gender

Comparing genders revealed no significant difference in age or BMI and no difference in mean OAHI or in the ratio of subjects with OAHI ≥ 1. There was no gender related difference in TAA in TST, in supine sleep or in REM sleep across all three levels and no difference in snoring.

### 3.6 Effects of Weight

Comparing normal‐weight and overweight subjects showed higher values for AHI and OAHI in the overweight group (Table 4) but other parameters were similar. Comparing normal‐weight and obese subjects, however, showed significantly higher values in the latter group for AHI and OAHI as well as for TAA as % of TST, supine sleep, and REM sleep across all three levels. There was also significantly increased snoring in obese subjects (Table 5).

**TABLE 5:** Comparison between normal‐weight, overweight and obese subjects.

	Normal‐weight (95% CI)	Overweight (95% CI)	Obese (95% CI)
*N*	71	23	19
Male gender, *N* (%)	50 (70)	14 (61)	12 (63)
Age in years (SD)	11.8 (±0.8)	11.6 (±0.8)	11.7 (±0.8)
AHI	0.6 (0.5–0.7)	0.9 (0.6–1.3)*	1.1 (0.4–1.9)*
OAHI	0.4 (0.3–0.5)	0.7 (0.4–0.9)*	1.0 (0.3–1.7)*
TAA > 40° in TST	3.8% (2.7–4.8)	4.4% (0.4–8.4)	7.7% (4.1–11.3)*
TAA > 80° in TST	0.9% (0.7–1.0)	1.0% (0.2–1.8)	2.3% (1.0–3.7)*
TAA > 120° in TST	0.3% (0.2–0.3)	0.4% (0.1–0.8)	0.7% (0.3–1.2)*
TAA > 40° in supine sleep	5.5% (4.0–6.9)	5.9% (1.1–10.7)	12.7% (6.0–19.5)*
TAA > 40° in REM sleep	11.1% (8.4–13.8)	10.5% (4.0–17.0)	19.2% (10.9–27.5)*
Snore in TST	1.9% (0.8–3.1)	0.9% (0.3–1.5)	6.8% (1.4–12.3)*
Snore in supine sleep	2.8% (1.1–4.5)	1.9% (−0.4–4.2)	10.0% (2.0–18.0)*

Abbreviations: AHI, apnea‐hypopnea index; OAHI, obstructive apnea‐hypopnea index; REM, rapid eye movement; TAA, thoracoabdominal asynchrony; TST, total sleep time.

* Significant at *p* < 0.05 compared to normal‐weight subjects.

### 3.7 Effects of Paediatric Sleep Questionnaire (PSQ) Score

Parent‐reported symptoms of SDB, using the PSQ, did not show a significant relationship with OAHI, TAA, gender or weight groups.

Looking only at the first six questions of the PSQ, pertaining to snoring and witnessed apneas, showed a higher score for the OAHI ≥ 1 group (mean score 0.19) than the OAHI < 1 group (mean score 0.07, *p* = 0.01) and a higher score for male (mean score 0.11) than for female gender (mean score 0.04, *p* = 0.03).

## 4 Discussion

In this study, our aim was to define the normal prevalence of TAA in healthy children without measurable OSA (with OAHI < 1). The range of normal TAA according to our study is defined by the 95th percentile of the healthy, non‐OSA group, containing all subjects of this group except the 5% with the most prevalent TAA values during sleep. For > 40° TAA this range contains values from 0% to 9.6% of TST. We suggest rounding up the cut‐off point to 10% for ease of use in clinical practice. The OSA group's mean value of 10.7% lies outside this range.

Comparing TAA in the non‐OSA group with that of subjects with OSA in our study showed that the latter group had a significantly higher prevalence of > 40° TAA during sleep. The difference was further illustrated by the fact that the 95% confidence intervals of the mean values of the two groups did not intersect. We did not find that the higher levels of TAA, 80° and 120°, added sensitivity to detecting differences between children with and without OSA, and propose that using the TAA level of 40° is adequate for analysis.

As expected, TAA was most prominent during REM sleep, since this is the sleep stage where striated muscle tone is minimal, the ribcage is more compliant and the risk for the collapse of the airways is the highest. TAA was also more prominent in supine sleep, where gravity can negatively affect airway patency.

Using the 95th percentile cut‐off to examine the whole cohort of 113 subjects enabled division into “normal TAA” and “abnormal TAA” subgroups. Comparison showed that the latter had a significantly higher mean OAHI score and more prevalent snoring, suggesting correlation between abnormal amounts of TAA and SBD.

The applicability of TAA analysis for better diagnosing OSA in children seems possible from our results, although further research is needed. However, our approach does not contribute to the scoring of conventional apnea–hypopnea events, which remain central to diagnosing OSA (Berry et al. 2012). Developing a method to correlate momentary TAA‐levels (as opposed to TAA prevalence during TST) with arousals on EEG would be a possible way forward to further highlight the effects of increased work of breathing on sleep quality and might increase the reliability of home sleep apnea tests (HSATs) in children. Combining apnea/hypopnea events and momentary TAA could then possibly be used for a more complete diagnosis of sleep disordered breathing. The utility of this analytic method is enhanced by the fact that the RIP bands required for its implementation are already incorporated in standard PSGs and in the simpler HSATs.

We suggest that assessing elevated TAA as % TST, as we did in our study, provides some new possibilities. It might be possible to more confidently exclude OSA in children with an OAHI slightly above normal, a finding frequently encountered in clinical practice. Currently, definitive exclusion of OSA in such cases would require a full PSG study. However, combining TAA analysis with HSATs might provide sufficient information to rule out OSA. Additionally, TAA analysis could assist in salvaging incomplete sleep studies which often necessitate repeat studies, with emotional strain and costs for those involved. The RIP bands are rarely the cause of failed studies (Montazeri et al. 2021) and using them to rule out significant TAA might obviate the need for a repeat study. Further research in the use of TAA in ruling out OSA is needed. Children with mildly elevated OAHI might also benefit from the addition of TAA analysis to their sleep studies, to aid in the decision of surgical treatment for OSA. In a study by Liu et al. (2017), elevated TAA was inversely related to quality of life in children with elevated OAHI. The study found that a reduction of tonsils and/or adenoids improved OAHI, TAA and quality of life. However, children assigned to watchful waiting who spontaneously improved in OAHI, but did not improve in TAA, had no improvement in quality of life. Consequently, more prevalent TAA may indicate a greater need for surgical intervention over watchful waiting. A caveat to the broader application of TAA analysis, and one that warrants further research, is the fact that we found elevated TAA to be more prevalent in supine sleep. Because of this, sleep position might need to be taken into account in TAA analysis as part of sleep study diagnostics and might further enhance the method's clinical applicability.

The mean prevalence of snoring in low‐OAHI subjects was only 1.5% of TST in our study. However, snoring was more prevalent in supine sleep and significantly increased in children with elevated OAHI and children with obesity as well as in those with abnormal TAA, as previously stated above. Increased snoring in children with elevated OAHI scores fit the results of parent‐reported snoring, using the PSQ questionnaire's first six questions that relate to snoring and apneas. Notably, when the PSQ was scored as a full 22‐item questionnaire it no longer showed concordance with elevated OAHI. This brings forth the question if the latter 16 questions of the PSQ have become less sensitive for the presence of OSA in our subjects only (10–13 years old children in Iceland) or children in general, and of a wider age span.

TAA has not, to our knowledge, been reported specifically for children with obesity before. The group of children with obesity had higher values in all measured variates of our study, including OAHI, with the prevalence of TAA doubling at all levels when compared to normal‐weight children. Comparison of the two groups showed that snoring was more than three times as prevalent in children with obesity and especially pronounced in supine sleep. The increase in snoring, OAHI, and TAA might all stem from the volume effect of adipose tissue on the upper airways, but factors such as increased weight of the chest and abdomen may factor in, as they do with adults. Being overweight, but not obese, had no measurable effect on TAA and snoring and related to only a slight increase in OAHI in our subjects.

Our study included children between the ages of 10–13 years. We propose that the results might be applicable from the age of 3 years since the increased TAA levels seen in infancy reach adult levels already at about 3 years of age (Kohyama et al. 2001). Adolescents above the age of 13 are traditionally sleep‐scored as adults, although that remains contentious (Tapia et al. 2008), and in our study, we did not address the scoring of adult sleep studies. Studying a wider age span is a logical step going forward to ascertain if similar values of TAA are seen in children and adolescents from the age of 3 to 18 years.

A possible limitation of the study is that children were not examined for upper airway obstruction such as enlarged tonsils or adenoids, which, in conjunction with symptoms of SDB, would have helped in further defining the normal or healthy group. This might have resulted in a lower normal TAA 95th percentile cut‐off. However, this could be seen as a strength for the use of TAA as a part of a screening tool for children with diverse symptoms of SDB. A limitation of our study population is that no underweight children were included, and the TAA prevalence is unknown in this group. A significant proportion (67%) of our study subjects were male. This could be an effect of the study design where a part of the group was invited to join based on parent‐reported snoring or apneas, and snoring is more often reported in boys than girls (Ersu et al. 2004). There was no gender difference with regards to OAHI, BMI or TAA at any level, supporting the use of our study results for both genders. Parent reported snoring was more prevalent in boys, but there was no objective difference between the genders. The study population was ethnically homogenous (data not shown), as the population of Iceland is in general, with a high proportion being of white Scandinavian background. The study's results should be viewed in light of that, and there is need of a study with a larger, more heterogenous population, with a wider age span and equal gender proportions. Such a study might lead to a broader applicability of TAA analysis for all children as a part of diagnosing OSA.

## 5 Conclusion

In our study we have aimed at defining the normal amount of thoracoabdominal asynchrony (TAA) during sleep in children between the ages of 10 and 13 years. We suggest that the results might be applicable to children down to 3 years of age since that is when thoracoabdominal movement reaches mature levels. We propose that having > 40° asynchrony for up to 10% of total sleep time is normal. Children with obesity had more symptoms of SDB and more TAA than children without obesity. With further research, more precise values of normal TAA could be defined in a wider age group. This might enable the incorporation of TAA into clinical practice and decrease the dependence on resource‐heavy and psychologically demanding in‐lab PSG in children.

## Author Contributions

**Sigurveig T. Sigurdardottir:** project administration, writing – review and editing. **Sigrun Helga Lund:** writing – review and editing, methodology. **Kristjan Dereksson:** conceptualization, investigation, funding acquisition, writing – original draft, formal analysis, project administration, writing – review and editing, visualization, validation, methodology. **Erna Sif Arnardottir:** resources, project administration, supervision, funding acquisition, writing – review and editing. **Sigridur Sigurdardottir:** investigation, writing – review and editing, software, formal analysis, data curation. **Michael V. Clausen:** writing – review and editing, project administration. **Erik A. Eklund:** funding acquisition, writing – review and editing, methodology, supervision. **Tryggvi Helgason:** writing – review and editing, methodology. **Agneta Markström:** writing – review and editing.

## Funding

This work was financially supported in part by NordForsk (NordSleep project 90458‐06111) via The Icelandic Centre for Research and Business Finland (5133/31/2018), the Icelandic Research Fund (no. 174067), the European Union's Horizon 2020 Research and Innovation Programme (grant no. 965417), the State Research Funding for university‐level health research, Kuopio University Hospital, Wellbeing Service County of North Savo (5041794, 5041803 and 5041812), the Landspitali University Hospital Science Fund 2019–2020 (no. A‐2019‐061 and A‐2020‐045), the Scientific Foundation of Respiratory Diseases, the Finnish Cultural Foundation‐North Savo Regional Fund, the Päivikki and Sakari Sohlberg Foundation, The Vilho, Yrjö and Kalle Väisälä Foundation, and in part by Tampere Tuberculosis Foundation. The birth cohort study was funded by the European Commission: (a) under the 6th Framework Programme (FOOD‐CT‐2005‐514,000) within the collaborative research initiative “EuroPrevall”; and (b) under the 7th Framework Programme (FP7‐KBBE‐2012‐6; grant agreement no. 312147) within the collaborative project “iFAAM”. Nox Medical supported the study by supplying PSG‐devices and consumables for the sleep studies.

## Conflicts of Interest

E. S. Arnardottir reports lecture honoraria from Nox Medical, Philips, ResMed, JazzPharmaceuticals, Linde Healthcare, Wink Sleep, Vistor (NovoNordisk) and Apnimed, as well as participation on the Philips Sleep Medicine and Innovation medical advisory board. However, all these financial disclosures are outside the scope of this work. T. Helgason declares owning shares in Nox Medical of monetary value less than 1 month's salary. No other conflicts of interest are declared.

## Supporting information


**Table S1:** Symptoms of sleep disordered breathing. Comparison of subjects with normal and abnormal thoracoabdominal asynchrony, using the 95th percentile cut‐off for 80° and 120° thoracoabdominal asynchrony.

## Data Availability

The data that support the findings of this study are available from the corresponding author upon reasonable request.
