# Supplementary material for: Thoracoabdominal Asynchrony in Healthy Children
Source: J Sleep Res. 2026 Jun 18;35(5):e70394. doi: 10.1111/jsr.70394 (PMC13619078; doi:10.1111/jsr.70394)
Supplement: Supplementary file 1 [file JSR-35-e70394-s001.docx]

**Supplemental material to the manuscript “Thoracoabdominal asynchrony in healthy children”**

**Supplemental table 1:** **Symptoms of sleep disordered breathing. Comparison of subjects with normal and abnormal thoracoabdominal asynchrony, using the 95^th^ percentile cut-off for 80° and 120° thoracoabdominal asynchrony**

|  | TAA >80° | | Significance |
| --- | --- | --- | --- |
| TAA group | **Normal (105)** | **Abnormal (8)** |  |
| OAHI (95% CI) | 0.5 (0.4-0.6) | 1.7 (0.0-3.4) | *P=0.00002* |
| Snore in TST (95% CI) | 2.2% (1.2-3.2) | 7.4% (-4.5-19.3) | *p=0.03* |
| Snore in supine sleep (95% CI) | 3.3% (1.7-4.9) | 10.3% (-5.8-26.4) | *p=0.05* |
|  | **TAA >120°** | | **Significance** |
| TAA group | **Normal (105)** | **Abnormal (8)** |  |
| OAHI (95% CI) | 0.5 (0.4-0.6) | 1.6 (-0.1-3.3) | *P=0.0001* |
| Snore in TST (95% CI) | 2.2% (1.2-3.2) | 6.9% (-5.2-19.0) | *p=0.04* |
| Snore in supine sleep (95% CI) | 3.4% (1.8-5.0) | 9.5% (-6.8-25.8) | *p=0.09 (NS)* |

*OAHI, obstructive apnea-hypopnea index. TAA, thoracoabdominal asynchrony. TST, total sleep time. CI, confidence interval. NS, not significant*
